# Estimating rice yield-related traits using machine learning models integrating hyperspectral and texture features

**DOI:** 10.3389/fpls.2025.1713014

**Published:** 2025-11-07

**Authors:** Yufen Zhang, Feifei Zhu, Kaiming Liang, Zhanhua Lu, Yibo Chen, Xuhua Zhong, Junfeng Pan, Chusheng Lu, Xiangyu Hu, Rui Hu, Meijuan Li, Xinyu Wang, Qunhuan Ye, Yuanhong Yin, Zhaowen Mo, Youqiang Fu

**Affiliations:** 1 Rice Research Institute, Guangdong Academy of Agricultural Sciences/Key Laboratory of Genetics and Breeding of High Quality Rice in Southern China (Co-construction by Ministry and Province), Ministry of Agriculture and Rural Affairs/Guangdong Key Laboratory of Rice Science and Technology, Guangzhou, China; 2 College of Agriculture, South China Agricultural University, Guangzhou, China

**Keywords:** hyperspectral, data dimensionality reduction, machine learning, texture features, rice

## Abstract

**Background:**

Rapidly estimating multiple trait indicators simultaneously, nondestructively, and with high precision is an important means of accurate diagnosis in modern phenomics. Increasing the accuracy of estimation models for rice yield-related trait indicators (leaf nitrogen concentration, LNC; leaf area index, LAI; aboveground biomass, AGB; and grain yield, GY) through a strategy of "spectral data + texture data + dimensionality reduction + machine learning" is highly important.

**Methods:**

Between 2022 and 2023, hyperspectral canopy images, the LNC, LAI, AGB, and GY were collected synchronously. Then, dimensionality reduction was performed on the preprocessed spectral data using the Pearson correlation coefficient method, the successive projections algorithm (SPA), and competitive adaptive reweighted sampling (CARS) to select sensitive wavelengths. Estimation models were constructed using artificial neural networks (ANNs), support vector machine regression, one-dimensional convolutional neural networks, and long short-term memory networks. By extracting the texture features corresponding to sensitive wavelengths, high-precision estimation models were constructed using a "spectral data + texture data + dimensionality reduction + machine learning" method.

**Results:**

SPA-ANN provided the best prediction for LNC (R^2^ = 0.82, RMSE = 3.68 g/kg) and LAI (R^2^ = 0.75, RMSE = 0.47), while CARS-ANN was optimal for AGB (R^2^ = 0.90, RMSE = 79.05 g/m2) and GY (R^2^ = 0.63, RMSE = 0.59 t/ha). Adding texture features increased R^2^ by up to 9.9% and reduced RMSE by up to 27.2%.

**Conclusion:**

The optimized method can significantly increase the accuracy of estimation models. The results provide a scientific basis and technical data for the precise diagnosis of rice yield-related traits.

## 1 Introduction

Rice (*Oryza sativa* L.) is one of China’s main food crops and plays an important role in ensuring the country’s food security and stabilizing economic development. Accurately monitoring and predicting multiple growth traits of rice is highly important for guiding healthy growth and precise fertilization management (Zhang et al., 2002). The leaf nitrogen concentration (LNC), leaf area index (LAI), aboveground biomass (AGB), and grain yield (GY) of rice are the most common indicators for monitoring and predicting the growth of high-yield and efficient rice (Wang et al., 2021). Research on the hyperspectral prediction of multiple growth indicators of rice is highly important for promoting the healthy growth of rice and increasing food security.

In recent years, owing to the efficient, accurate, objective, and nondestructive characteristics of hyperspectral remote sensing technology, scholars worldwide have conducted extensive research on monitoring various biological indicators of field crops using hyperspectral technology and have achieved useful results (Onoyama et al., 2018; Xu et al., 2023; Zhang et al., 2024). These studies often use the full spectrum for modelling. However, the predictive performance of the models is somewhat affected by the presence of a large amount of redundant information in the spectrum (Gao, 1993; Kuusk, 2001). When conducting hyperspectral analysis, the high spectral values generally range from 400 to 2500 nm, which leads to very high data dimensionality. Therefore, reducing the dimensionality of spectral data and identifying sensitive wavelengths are the primary conditions for improving the efficiency of model operation, simplifying the model structure, and increasing model stability (Barman and Patra, 2020).

Huan et al. (Huan et al., 2021) used four dimensionality reduction algorithms—CARS, variable combination population analysis, Monte Carlo variable combination population analysis, and automatic weighted variable combination population analysis (AWVCPA)—to extract spectral feature wavelengths for wheat protein content. They combined these methods with partial least squares regression (PLSR) to construct a quantitative detection model for wheat protein, with the AWVCPA-PLSR model yielding the best performance. Wang et al. (Wang et al., 2020) used the SPA to select sensitive wavelengths for above-ground biomass. The SPA-PLSR model for estimating and validating the aboveground biomass of winter wheat achieved high accuracy. Tong et al. (Tong et al., 2020) used mathematical transformation and discrete wavelet transform algorithms to process and analyze the spectral data of passion fruit leaves. They utilized the PCC method to extract sensitive wavelengths. The results showed that the PCC–PLSR model for estimating the chlorophyll content in passion fruit leaves was superior. Therefore, there are significant differences in dimensionality reduction methods with different monitoring targets and trait indicators.

Machine learning is an interdisciplinary field involving multiple areas, such as statistics, probability theory, convex analysis, and algorithmic complexity theory (de Castro et al., 2020). Traditional linear regression methods have limitations in terms of model fit, often resulting in low model accuracy. With the development of machine learning technology, algorithms such as artificial neural network (ANN), support vector regression (SVR), and random forest (RF) have been widely used to estimate biochemical crop parameters (Kganyago et al., 2021; Jabed and Azmi Murad, 2024). Mao et al. (Mao et al., 2024) obtained hyperspectral remote sensing data for winter wheat and used four mainstream machine learning algorithms to predict wheat yield. They reported that the transfer learning model with multiple random ensembles has advantages in improving the Pearson correlation coefficient. Cheng et al. (Cheng et al., 2017) established estimation models for the soil and plant analyzer development (SPAD) values of apple leaves on the basis of univariate regression models and SVR models. The research results showed that the SVR model had greater estimation accuracy for the SPAD values of apple leaves. Therefore, the most suitable model algorithm is different for different crops or different trait indicators. Moreover, single-spectrum data may not provide comprehensive information about the growth status of crops. Spectral data mainly reflect the biochemical characteristics of crops, whereas texture features include information about their spatial structure and morphology.

In recent years, the number of studies estimating crop growth conditions on the basis of unmanned aerial vehicle imagery, combined with spectral and texture features, has gradually increased (Liu et al., 2019; Yue et al., 2019). Research has shown that integrating vegetation indices with texture features can effectively increase the estimation accuracy of various growth parameters, such as the nitrogen nutrition index (Yang et al., 2020), biomass (Liu et al., 2018), and chlorophyll (Chen et al., 2019) content. Therefore, the effective integration of spectral and texture features is of significant importance for enhancing the accuracy of estimation models for various crop trait parameters.

In summary, in constructing spectral estimation models for crop biochemical parameters, different data dimensionality reduction techniques and machine learning algorithms each have advantages, but there are still significant challenges in selecting the best methods. However, studies on integrating spectral data with texture features to estimate rice yield-related traits, based on the optimal combination of dimensionality reduction techniques and machine learning algorithms, have rarely been reported. In this study, the widely cultivated high-quality indica varieties “Meixiangzhan No. 2” and “Nanjingxiangzhan” in South China were used as experimental materials. Using a multirotor drone M300 RTK equipped with an X20P airborne hyperspectral imager, the spectral data of rice were collected at four key growth stages between 2022 and 2023. Different combinations of dimensionality reduction and modelling methods were examined, and model evaluation indicators were used to construct high-precision estimation models for rice yield-related traits that combine spectral data, texture features, dimensionality reduction, and machine learning on the basis of the best combination model. This further improves the estimation capability of the models. The results provide a theoretical basis and scientific guidance for the precise phenotypic diagnosis and prediction of yield-related traits of high-quality indica rice varieties in South China.

## 2 Materials and methods

### 2.1 Experimental materials and soil physicochemical properties

**Figure 1:**
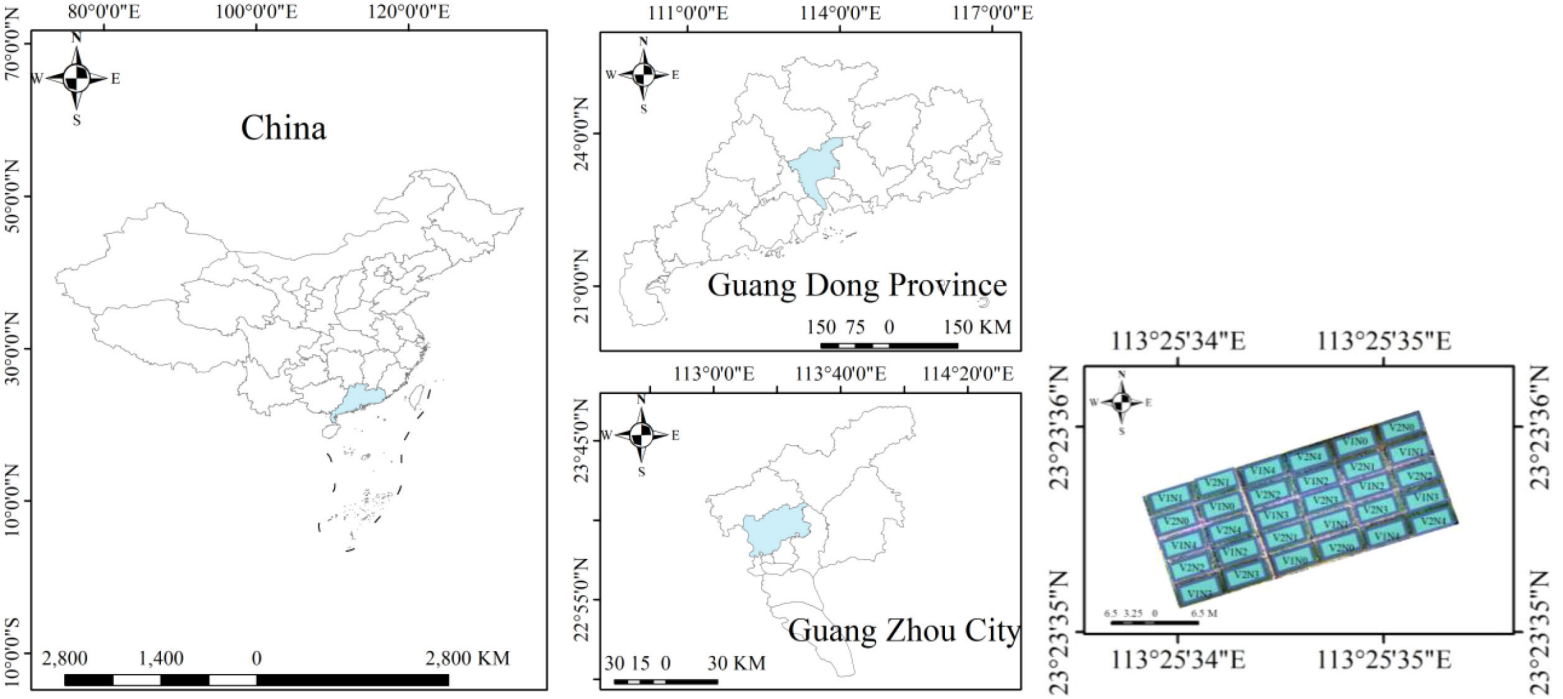
Overview of the experimental site. V1 and V2 represent “Meixiangzhan No. 2” and “Nanjingxiangzhan”, respectively. N0, N1, N2, N3, and N4 represent treatments of 0, 60, 120, 180, and 240 kg/ha, respectively.

The experimental rice varieties used were the high-quality indica rice varieties “Meixiangzhan No. 2” (V1) and “Nanjingxiangzhan” (V2), which are widely cultivated in South China. The experiment was conducted at the Guangdong Academy of Agricultural Sciences experimental base in Zhongluotan town, Baiyun district, Guangzhou city, Guangdong province, during the early and late seasons of 2022 and the early season of 2023 (**Figure 1**). The physicochemical properties of the experimental field soil were as follows: pH, 5.95; organic matter, 22.48 g/kg; total nitrogen, 1.29 g/kg; total phosphorus, 0.42 g/kg; total potassium, 8.43 g/kg; alkaline hydrolysable nitrogen, 58.03 mg/kg; available phosphorus, 6.49 mg/kg; and readily available potassium, 47.00 mg/kg.

### 2.2 Experimental design

The experiment used a split-plot design, with nitrogen levels as the main plot and varieties as the subplot. Five nitrogen fertilizer levels were considered: 0, 60, 120, 180, and 240 kg N/ha. Transplanting was performed manually using a line-drawing method, with a planting density of 20 cm × 20 cm, two seedlings per hill, and three replicates. Nitrogen fertilizer was applied in the form of urea, and phosphorus and potassium fertilizers were applied as superphosphate and potassium chloride, respectively. Nitrogen fertilizer was applied at a ratio of basal fertilizer: tillering fertilizer: panicle fertilizer=5:2:3. Basal fertilizer was applied one day before transplanting, tillering fertilizer was applied 15 days after transplanting, and panicle fertilizer was applied at the panicle initiation (PI) stage. Phosphorus and potassium fertilizers were applied at standard rates of 54 kg P_2_O_5_/ha and 144 kg K_2_O/ha, respectively. All the phosphorus fertilizer was applied as basal fertilizer, and half of the potassium fertilizer was applied as basal fertilizer, with the other half applied as panicle fertilizer. The field ridges of each plot were wrapped with plastic film (PVC) to prevent fertilizer runoff between plots. Field management was conducted according to the “three controls” (Zhong et al., 2007) fertilization technique for rice, which optimizes nitrogen application to control fertilization, tillering, and pests. Strict prevention and control of pests and diseases. High spectral data of the canopy and data for four agronomic traits (LNC, LAI, AGB, and GY) were collected at the heading stage (HD) in the early season of 2022 on June 16, the panicle initiation stage (PI) in the late season of 2022potassium fertilizers were applied at standard rates of 54 kg on September 16, the heading stage (HD) in the late season of 2022 on October 12, and the panicle initiation stage (PI) in the early season of 2023 on May 15.

### 2.3 Experimental methods

#### 2.3.1 Hyperspectral data acquisition and preprocessing

The hyperspectral rice canopy data were acquired with an M300 RTK (DJI, China) multirotor drone equipped with an X20P (Cubert, Germany) airborne hyperspectral imager. The spectral range of the imager is 350 to 1000 nm, with a resolution of 4 nm and 164 effective bands. The drone flies at a height of 50 meters. The measurement time ranged from 10:00 to 14:00 Beijing time, and the weather was clear. Through image stitching, radiometric calibration, atmospheric correction, orthorectification, image fusion, geometric correction, and band normalization, the spectral average value of the region of interest (ROI) in each plot was extracted using an ROI tool to determine the original spectral reflectance of each plot. To address the noise bands caused by environmental factors and increase the prediction accuracy of the estimation model, the original spectra were processed using the Savitzky–Golay convolution smoothing algorithm (Wu et al., 2014). The Savitzky–Golay convolution smoothing algorithm uses a polynomial to perform a polynomial least squares fit on the data within a moving window. Essentially, it is a weighted averaging method that emphasizes the central role of the center point (Chu et al., 2004). This enables effective noise reduction while preserving the original spectral information well (Li SL. et al., 2024). After parameter optimization, the window_length value for the Savitzky–Golay convolution smoothing method was set to 9, and the number of polynomials was set to 2.

#### 2.3.2 Data dimensionality reduction methods

Through comparative analysis, we adopted three of the most representative dimensionality reduction methods for analysis: PCC, SPA, and CARS. PCC is an analysis method based on the partial least squares regression model. This method primarily involves calculating and analyzing the correlation between the spectral data corresponding to each band and the biometric data of the rice to screen out combinations of feature bands with higher correlations (Yang et al., 2015). SPA uses vector projection to select the minimum number of spectral variables, addressing issues such as information overlap and collinearity in the spectra. This reduces the number of variables used in modelling, thereby increasing the modelling efficiency (Araújo et al., 2001). CARS simulates Darwin’s evolutionary concept of “survival of the fittest.” Through adaptive reweighted sampling techniques, wavelengths with larger absolute regression coefficients as calculated by partial least squares regression are selected. Cross–validation is then used to choose the combination set with the lowest root mean square error (Li et al., 2009).

#### 2.3.3 Hyperspectral image texture feature extraction

Using the grey–level co–occurrence matrix method in ENVI 5.3, the texture features of the selected sensitive bands are extracted with a 3×3 resolution window size, the spatial correlation matrix offsets X and Y default to 1. These include the mean, variance, homogeneity, contrast, dissimilarity, entropy, second moment, and correlation (Yang et al., 2020). The Regions of Interest (ROI) for each plot were delineated on the texture feature images of each band using the Region of Interest (ROI) tool, the texture values of those regions can be extracted. A correlation analysis was conducted between the extracted texture values and various biophysical parameters. The two texture values showing the strongest correlations with the biophysical parameters were selected as inputs.

#### 2.3.4 Biological indicators

After the spectral measurements were performed, 12 representative rice plants were randomly selected from the experimental plots. The root systems were removed. At the PI stage, the stems and leaves were separated, and at the HD stage, the stems, leaves, and panicles were separated. A leaf area meter was used to measure the leaf area (S). The stems, leaves, and panicles were then placed in an oven at 105°C for blanching for 30 min, followed by drying at a constant temperature of 75°C until a constant weight was reached. The dry weights were recorded separately (w1, w2, w3).

Leaf Nitrogen Concentration (LNC, g/kg): After grinding, the nitrogen concentration in the rice leaves was determined using the Kjeldahl method (Yu et al., 2022).

Leaf Area Index (LAI): This index is calculated via Equation 1.

(1) $$LAI=\frac{S}{C}\times D$$

In Equation 1, *C* represents the number of sampled holes, and *D* represents the planting density, which is the number of rice holes per unit area.

Aboveground Biomass (AGB, g/m This calculation is based on the planting density at the sampling points and the dry weight of the sampled rice, as shown in Equation 2.

(2) $$AGB=\frac{\left(w1+w2+w3\right)}{C}\times D$$

In Equation 2, *C* is the number of sampled holes, and *D* is the planting density.

Grain Yield (GY, t/ha): During the maturity stage, 125 rice plants (5 m²) were harvested from each plot to measure yield. The paddy material was air–dried, and approximately 100 g was dried at 105°C for 48 hours to determine the moisture content. The paddy material was then converted to a yield with a moisture content of 14% (Jing et al., 2022).

#### 2.3.5 Model construction and validation

In this experiment, four algorithms are used to establish hyperspectral prediction models for the four rice agronomic traits at the panicle initiation, heading and maturation stages (**Table 1**): ANN, SVR, one–dimensional convolutional neural network (1DCNN), and long short–term memory (LSTM).

**Table 1:** Statistics of measured yield-related traits at various rice growth stages.

Collected indicators	Sampling time	Growth stages	Sample size	Minimum value	Maximum value	Mean
LNC(g/kg)	2022.06.16	HD	30	13.902	19.720	17.282
	2022.09.16	PI	30	15.559	23.036	20.218
	2022.10.12	HD	30	14.913	24.557	19.220
	2023.05.15	PI	30	27.829	51.673	38.570
LAI	2022.06.16	HD	30	1.609	5.031	3.170
	2022.09.16	PI	30	1.410	3.529	2.626
	2022.10.12	HD	30	1.871	5.445	3.597
	2023.05.15	PI	30	1.299	3.392	2.273
AGB(g/m^2^)	2022.06.16	HD	30	462.521	852.333	669.876
	2022.09.16	PI	30	222.458	409.146	316.796
	2022.10.12	HD	30	580.875	1039.479	791.457
	2023.05.15	PI	30	115.438	268.458	197.110
GY(t/ha)	2022.07.12	MA	30	3.810	6.485	5.300
	2022.11.16	MA	30	3.929	7.410	5.753
	2023.07.10	MA	30	3.363	7.227	5.538

LNC, LAI, AGB and GY represent the leaf nitrogen concentration, leaf area index, aboveground biomass and grain yield, respectively. PI, HD and MA represent the panicle differentiation, heading and maturation stages, respectively.

The ANN was first proposed by psychologist McCulloch and mathematician Pitts in 1943. They constructed the M–P model, which combines the working principle of neurons with logical operations, thereby laying the theoretical foundation for the development of ANNs (Mcculloch and Pitts, 1943). The ANN is a computational model that mimics the structure and function of the neural network of the human brain. It consists of many nodes (or “neurons”), which are typically arranged in layers, such as the input layer, hidden layers, and output layer. Each node receives input from the nodes in the previous layer, performs a weighted sum, and then generates an output through a nonlinear activation function, which is passed to the next layer. ANNs learn the complex relationships and patterns of input data by adjusting the connection weights between neurons (Lussier et al., 2020). The main parameters of the ANN—”activation”, “alpha”, “hidden_layer_sizes”, “learning_rate”, “max_iter”, “momentum”, “solver”, and “tol”—were set to relu, 0.0001, 100, adaptive, 200, 0.7, adam, and 0.00001, respectively.

SVR (Bakhshipour and Jafari, 2018) is widely applied in the fields of machine learning, artificial intelligence, and big data. It was originally designed to address binary classification problems. SVR works by finding the regression plane to which all the data points in a set are closest. The algorithm features a kernel function that allows it to flexibly address various nonlinear regression problems (Zhang J. et al., 2021). The main parameters of the SVR—”kernel”, “degree”, “gamma”, “coef0”, “tol”, “C”, “Epsilon”, “shrinking”, “cache_size”, “verbose”, and “max_iter”—were set to rbf, 3, auto, 0.0, 0.001, 1.0, 0.1, True, 200, False, and −1, respectively.

The prototype of a 1DCNN, LeNet–5, was first proposed in 1998 (Lecun et al., 1998). This model is based on a convolutional and pooling network structure and is trained using the backpropagation (BP) algorithm. Convolutional neural networks can automatically extract data features without manual intervention and possess strong robustness and fault tolerance, making them easy to train and optimize. Additionally, they have abilities such as local perception, parameter sharing, and –multilevel feature abstraction (Hu et al., 2024). The main parameters of the 1DCNN—”in_channels”, “out_channels”, “kernel_size”, “padding”, and “num_epochs”—were set to 1, 16, 3, 1, and 10, respectively.

LSTM is a type of recurrent neural network (RNN) with memory capabilities that was originally proposed by Hochreiter et al. and is specifically designed for processing time series data (Hochreiter and Schmidhuber, 1997). It addresses the issues of gradient vanishing and gradient explosion that occur in traditional RNNs when processing long time series, particularly the problem of gradient vanishing (Wang P. et al., 2023). The main parameters of the LSTM—”input_size”, “output_size”, “hidden_size_temp”, “num_layer_temp”, and “drop_temp”—were set to 3, 1, 64, 1, and 0.4, respectively.

**Figure 2:**
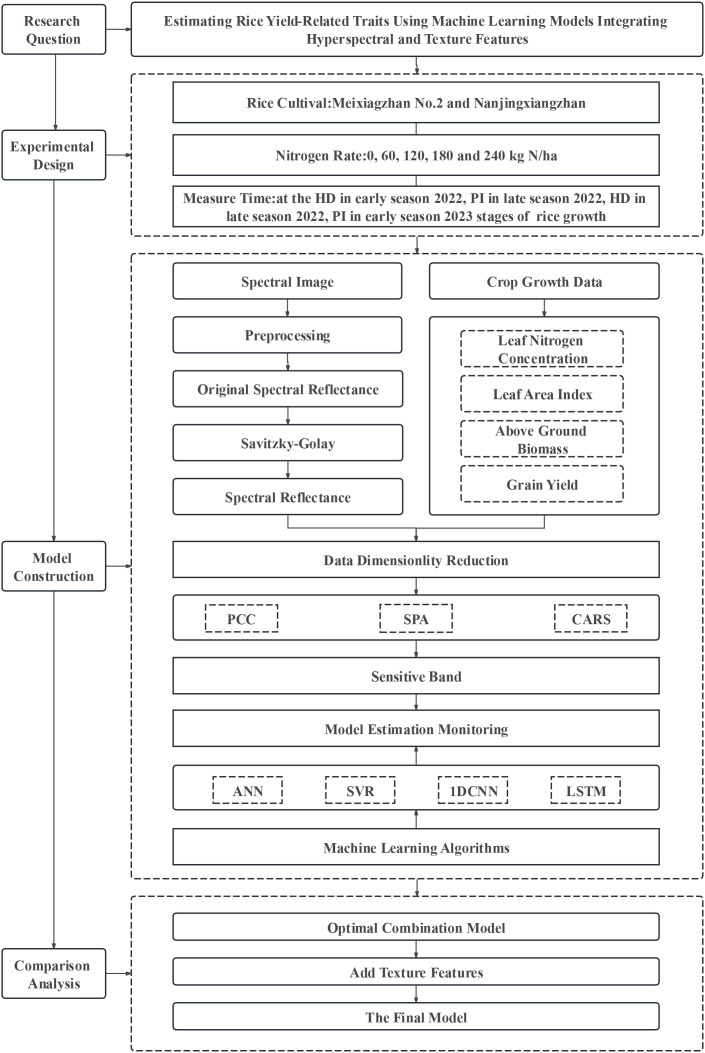
Technology roadmap. PCC, SPA, and CARS represent the Pearson correlation coefficient, successive projections algorithm, and competitive adaptive reweighted sampling, respectively; ANN, SVR, 1D CNN, and LSTM represent –the artificial neural network, support vector regression, the 1D convolutional neural network, and long short–term memory, respectively. PI and HD represent the panicle differentiation and heading stages, respectively.

The model construction process is shown in **Figure 2**.

A total of 120 samples were collected across the four periods. During modelling, 70% of the samples were randomly selected for model building, and 30% of the samples were used for model accuracy validation. All the parameter estimation models were evaluated using the coefficient of determination (R^2^) and the root mean square error (RMSE) to assess model accuracy (Niu et al., 2021). Here, R^2^ is used to evaluate the model’s fit, with values closer to 1 indicating a better fit; RMSE is used to evaluate the model’s stability, with smaller values indicating greater stability. The calculation formulas for R^2^ and RMSE were shown in Equations 3, 4, respectively (Li et al., 2022; Li et al., 2023).

(3) $$R^{2}=1-(\Sigma_{i=1}^{n}{\left({\hat{y}}_{i}-{\bar{y}}_{i}\right)}^{2})/(\Sigma_{i=1}^{n}{\left(y_{i}-{\bar{y}}_{i}\right)}^{2})$$

(4) $$RMSE=\sqrt{\frac{1}{n}\times ({\sum_{i=1}^{n}\left({\hat{y}}_{i}-{\bar{y}}_{i}\right)}^{2}})$$

where *n* is the number of samples, ${\hat{y}}_{i}$ is the predicted value, ${\bar{y}}_{i}$ is the mean value, and $y_{i}$ is the actual value.

### 2.4 Statistical methods

Hyperspectral images were mosaicked via PhotoScan 2.0.2.16102 software and were radiometrically corrected, preprocessed, and texture extracted via ENVI 5.3 software. The experimental data were analyzed using Excel 2019 and SPSS 27.0, and graphs were created using Origin 2022 and ArcMap 10.8. Data dimensionality reduction and model training were conducted using Python 3.6 software.

## 3 Results and analysis

### 3.1 Spectral reflectance smoothing

**Figure 3:**
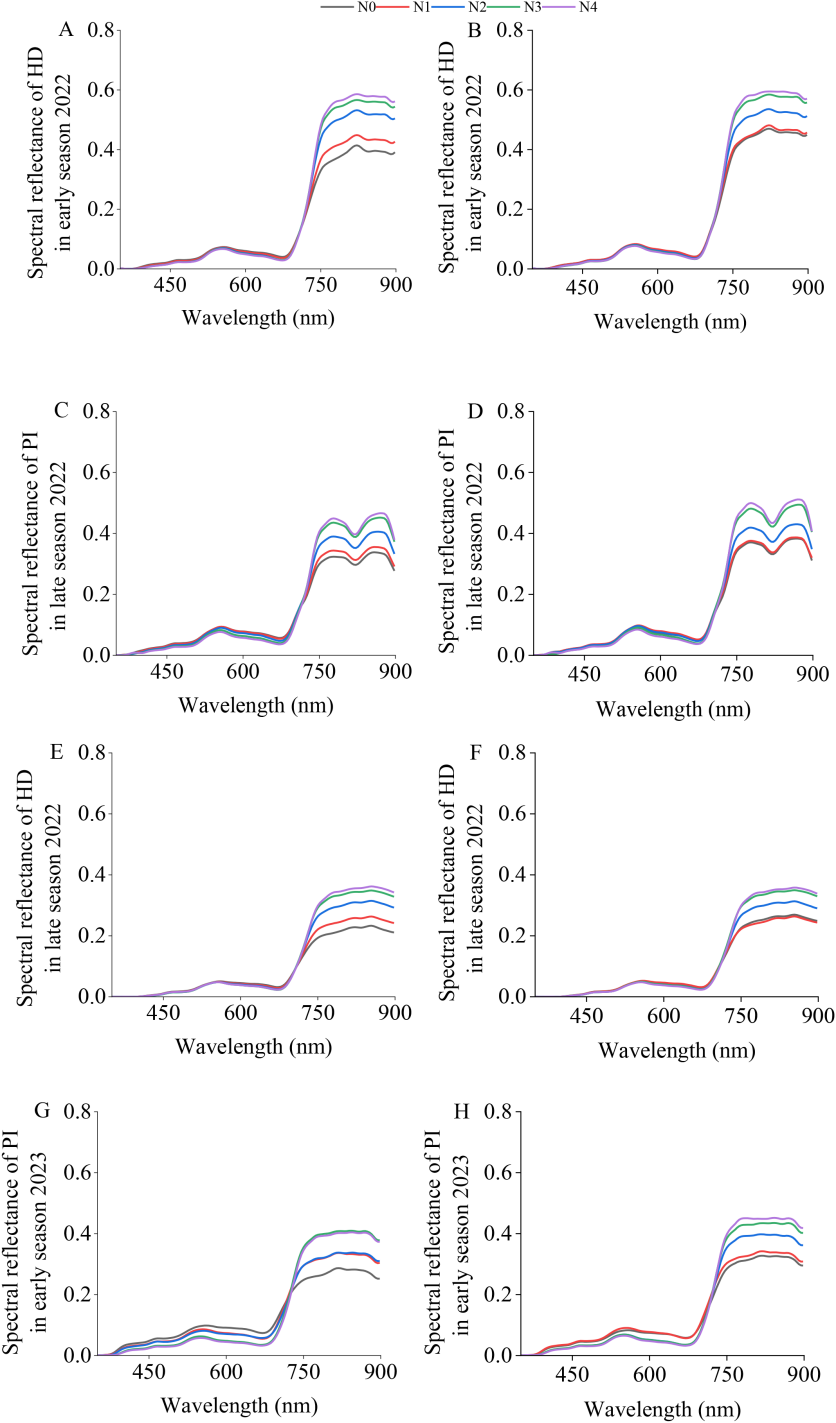
SG–smoothed canopy spectral reflectances of “Meixiangzhan No. 2” **(A, C, E, G)** and “Nanjingxiangzhan” **(B, D, F, H)** at various growth stages. The spectral range displayed is limited to 400–900 nm to exclude the noisy regions at the spectral extremes (particularly beyond 900 nm) of the X20P sensor, ensuring a clear presentation of the reliable spectral data used for analysis.

**Figure 3** shows the spectral reflectance after SG smoothing, which effectively reduces the “jagged” appearance of the spectral reflectance. The canopy spectral reflectances of the two varieties during the four sampling periods showed consistent trends. Between 350 nm and 720 nm, the canopy spectral reflectance showed no significant differences. Between 720 nm and 898 nm, the spectral reflectance increased with increasing nitrogen application.

### 3.2 Biological indicators

**Figure 4:**
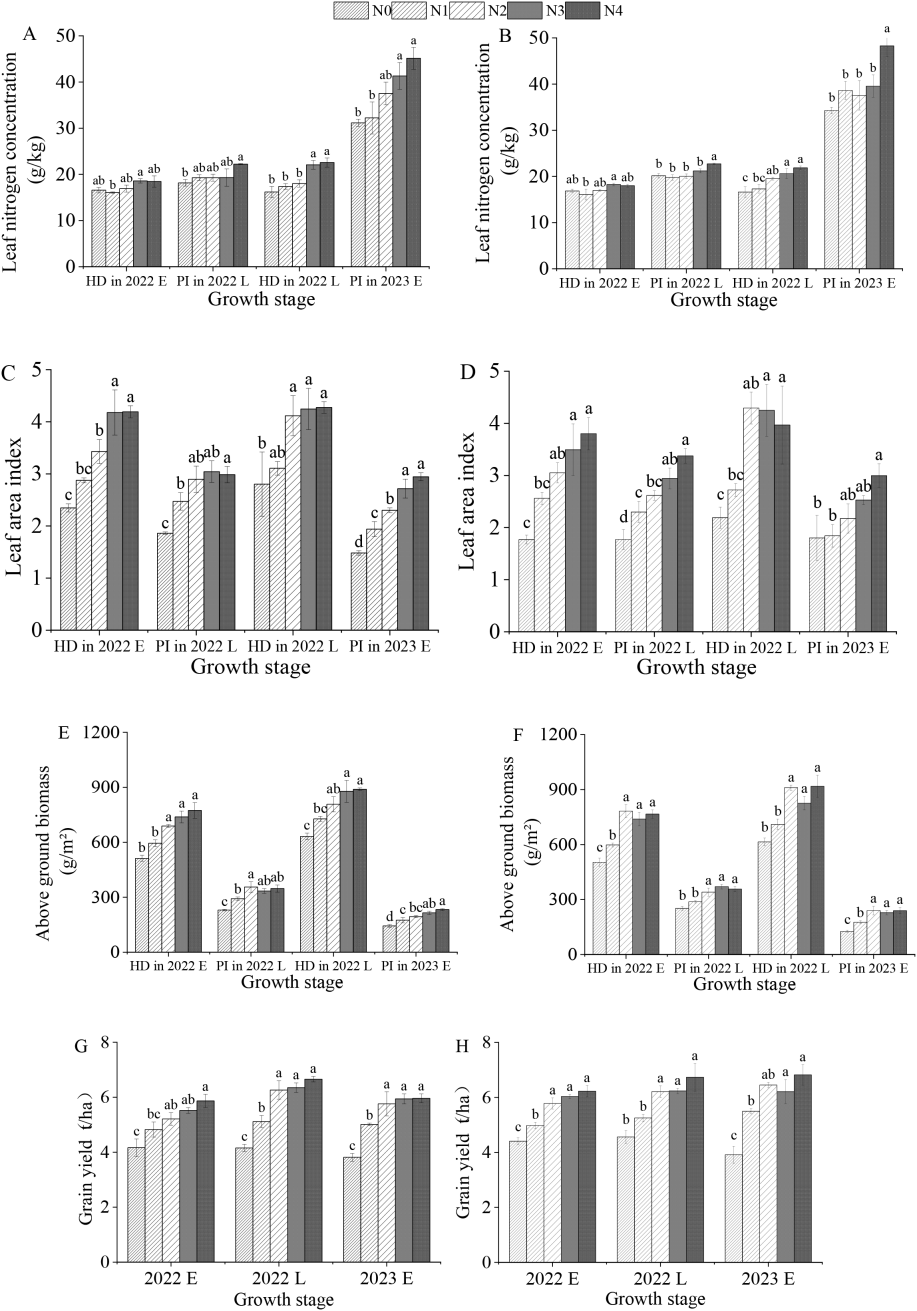
Effects of different nitrogen fertilizer levels on the leaf nitrogen concentration, leaf area index, –aboveground biomass, and yield of “Meixiangzhan No. 2” **(A, C, E, G)** and “Nanjingxiangzhan” **(B, D, F, H)** at various growth stages. HD in 2022 E, PI in 2022 L, HD in 2022 L, and PI in 2023 E represent the heading stage in the early season of 2022, the beginning of panicle differentiation in the late season of 2022, the heading stage in the late season of 2022, and the beginning of panicle differentiation in the early season of 2023, respectively; N0, N1, N2, N3, and N4 represent treatments of 0, 60, 120, 180, and 240 kg/ha, respectively; Lowercase letters for the same growth stage under different nitrogen fertilizer levels indicate significant differences at the p < 0.05 level.

As shown in **Figure 4**, with increasing nitrogen application, the LNC, LAI, AGB, and GY of rice gradually increased. For both the “Meixiangzhan No. 2” and the “Nanjingxiangzhan” rice varieties, when the nitrogen application reached 120 kg/ha, there was no significant difference in GY among the N2, N3, and N4 treatments (P< 0.05).

### 3.3 Correlations of trait indicators

**Figure 5:**
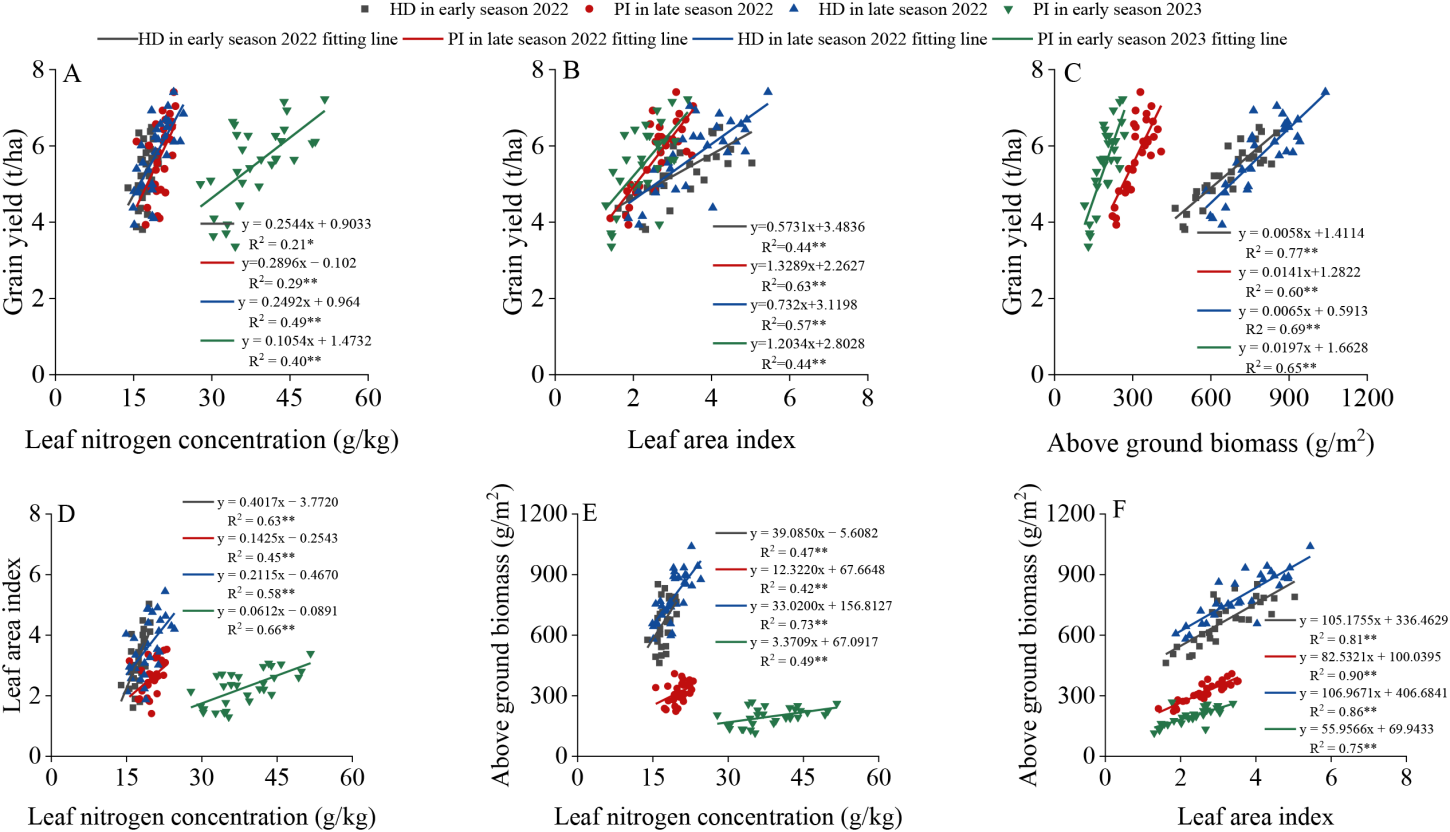
Correlations between leaf nitrogen concentration **(A)**, leaf area index **(B)**, aboveground biomass **(C)** and yield at different growth stages of rice, as well as correlations between leaf nitrogen concentration and leaf area index **(D)**, leaf nitrogen concentration and aboveground biomass **(E)**, and leaf area index and aboveground biomass **(F)**. PI and HD represent the PI stage and heading stage, respectively. “*” and “**” indicate significant correlations at the P<0.05 and P<0.01 levels, respectively.

As shown in **Figure 5**, LNC, LAI, and AGB all exhibited significant positive correlations with GY, with the strength of these relationships expressed as the coefficient of determination (R²), which is the square of the Pearson correlation coefficient (r). Among the correlations of AGB, LNC, and LAI with GY, the correlation between AGB and GY was the highest, with R^2^≥0.60, followed by LAI, with R^2^≥0.44. Regarding the correlations among AGB, LNC, and LAI, the correlation between AGB and LAI was the highest, with R^2^≥0.75.

### 3.4 Feature band selection

**Figure 6:**
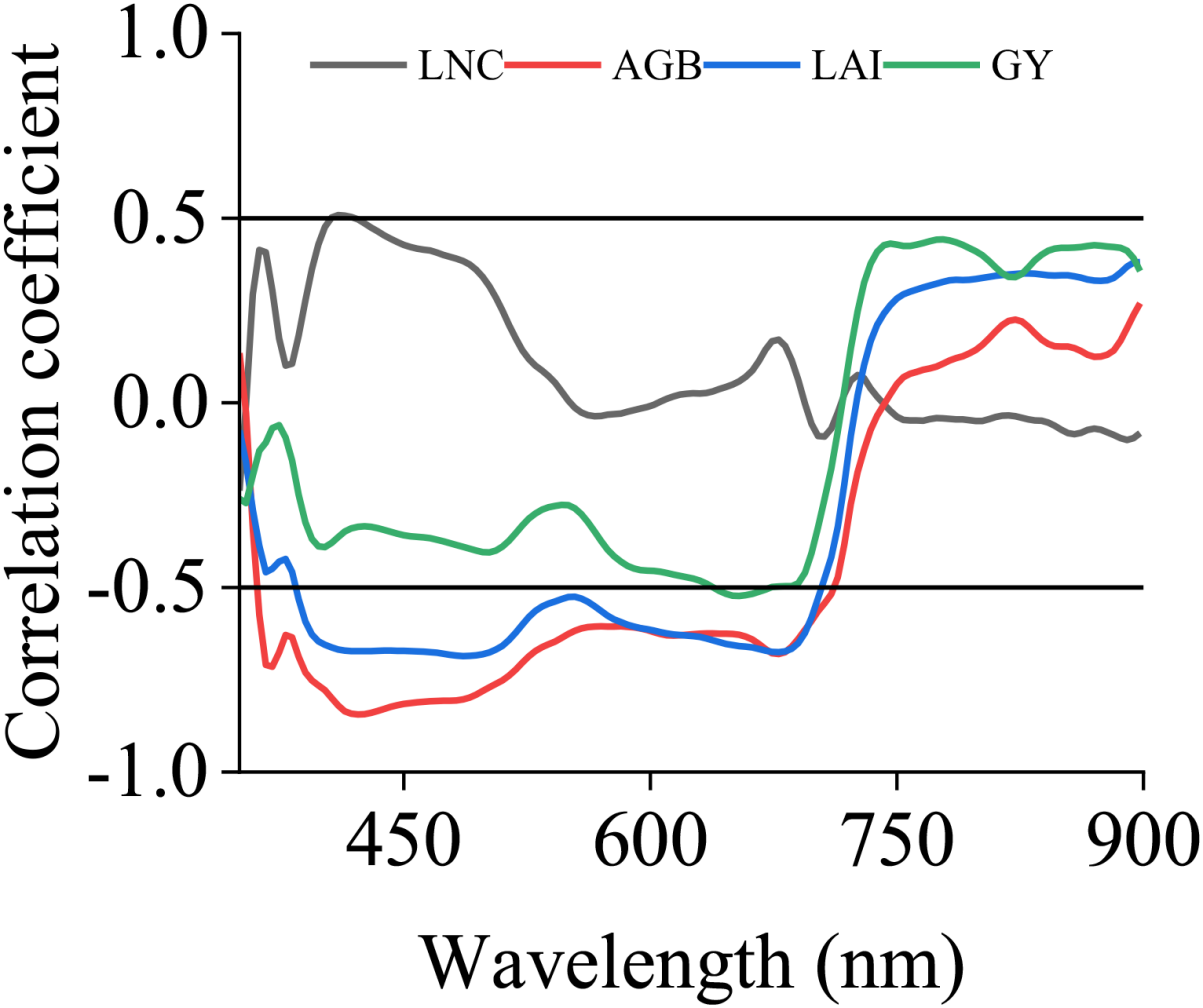
Correlation coefficients between spectral reflectance and biological indicators. LNC, LAI, AGB and GY represent the leaf nitrogen concentration, leaf area index, aboveground biomass and grain yield, respectively.

A correlation analysis was conducted between canopy spectral reflectance and various trait indicators. When the absolute value of the correlation coefficient was greater than 0.5, it was considered to indicate strong correlation. As shown in **Figure 6**, when the PCC dimensionality reduction method was used, the bands strongly correlated with LNC were found to be 406, 410, 414, 418, and 422 nm. Among them, the band with the strongest correlation to LNC was 410 nm, with a correlation coefficient of 0.508. AGB had a strong correlation in the range of 366 nm to 694 nm, with the strongest correlation occurring at 422 nm, where the correlation coefficient reached 0.845. LAI had a strong correlation in the range of 454 nm to 502 nm, as well as at 425 nm and 426 nm, with the strongest correlation occurring at 486 nm, where the correlation coefficient reached 0.686. AGB had a strong correlation in the range of 366 nm to 694 nm, with the strongest correlation occurring at 422 nm, where the correlation coefficient reached 0.845. GY had a strong correlation in the range of 642 nm to 670 nm, with the strongest correlation occurring at 654 nm, where the correlation coefficient reached 0.523. In addition, SPA and CARS were used to screen sensitive bands. Ultimately, the top 3 bands with the strongest correlations obtained from PCC and CARS and the top 5 bands obtained from SPA were selected as sensitive bands. The results are shown in **Table 2**.

**Table 2:** Sensitive bands of rice yield–related traits after dimensionality reduction.

Dimensionality reduction methods	LNC	LAI	AGB	GY
PCC	410nm、414nm、418nm	422nm、486nm、678nm	370nm、422nm、678nm	650nm、654nm、658nm
SPA	398nm、410nm、478nm、498nm、590nm	350nm、378nm、394nm、486nm、890nm	402nm、422nm、450nm、718nm、898nm	630nm、654nm、658nm、690nm、742nm
CARS	406nm、410nm、414nm	402nm、674nm、678nm	366nm、370nm、418nm	350nm、398nm、898nm

LNC, LAI, AGB and GY represent the leaf nitrogen concentration, leaf area index, aboveground biomass and grain yield, respectively. PCC, SPA and CARS represent person correlation coefficient, successive projections algorithm, and competitive adaptive reweighted sampling, respectively.

### 3.5 Estimation modelling and analysis

Three dimensionality reduction methods, PCC, SPA, and CARS, were used for sensitive band selection. The selected sensitive bands were used as feature inputs to construct estimation models for LNC, LAI, AGB, and GY using four machine learning algorithms: ANN, SVR, 1DCNN, and LSTM. The resulting model R² and RMSE values are shown in **Table 3**. When PCC was used for sensitive band selection, the estimation of AGB yielded the best results. The model using the ANN algorithm had the highest precision and stability, with an R² of 0.89 and an RMSE of 86.25 g/m^2^. When SPA was used for sensitive band selection, the estimation of LNC, LAI, and AGB yielded the best results. In this case, the model using the ANN algorithm had the highest precision and stability, with R² values of 0.82, 0.75, and 0.90 for LNC, LAI, and AGB, respectively, and RMSE values of 3.68 g/kg, 0.47, and 81.29 g/m^2^, respectively. When CARS was used for sensitive band selection, the estimations of LAI, AGB, and GY yielded the best results. For LAI, the model using the SVR algorithm had the highest precision and stability, with an R² of 0.62 and an RMSE of 0.54. For AGB and GY, the models using the ANN algorithm had the highest precision and stability, with R² values of 0.90 and 0.63 and RMSE values of 79.05 g/m^2^ and 0.59 t/ha, respectively. In summary, among the three data dimensionality reduction methods and four modelling approaches, the model constructed using SPA–ANN had high precision and good stability for estimating LNC and LAI. For estimating AGB and GY, the model built using CARS–ANN was the best.

**Table 3:** Model accuracy.

Dimensionality reduction methods	Machine learning algorithms	LNC		LAI		AGB		GY	
		R²	RMSE	R²	RMSE	R²	RMSE	R²	RMSE
PCC	ANN	0.31	7.63	0.58	0.56	0.89	86.25	0.26	0.79
	SVR	0.28	8.30	0.59	0.56	0.85	99.99	0.28	0.78
	1DCNN	0.27	8.02	0.53	0.62	0.85	98.95	0.29	0.75
	LSTM	0.26	7.81	0.50	0.66	0.73	141.31	0.28	0.91
SPA	ANN	0.82	3.68	0.75	0.47	0.90	81.29	0.54	0.65
	SVR	0.73	5.42	0.67	0.56	0.85	97.02	0.47	0.69
	1DCNN	0.79	4.60	0.68	0.53	0.87	94.57	0.50	0.69
	LSTM	0.28	8.22	0.48	0.66	0.74	130.59	0.40	0.79
CARS	ANN	0.35	8.01	0.61	0.58	0.90	79.05	0.63	0.59
	SVR	0.28	8.27	0.62	0.54	0.87	95.15	0.53	0.67
	1DCNN	0.27	8.01	0.58	0.58	0.84	107.07	0.43	0.72
	LSTM	0.28	7.45	0.48	0.69	0.70	148.19	0.10	0.95

LNC, LAI, AGB and GY represent the leaf nitrogen concentration, leaf area index, aboveground biomass and grain yield, respectively. PCC, SPA and CARS represent person correlation coefficient, successive projections algorithm, and competitive adaptive reweighted sampling, respectively. ANN, SVR, 1DCNN and LSTM represent artificial neural network, support vector regression, the 1D convolutional neural network and long short–term memory, respectively.

### 3.6 Model validation

**Figure 7:**
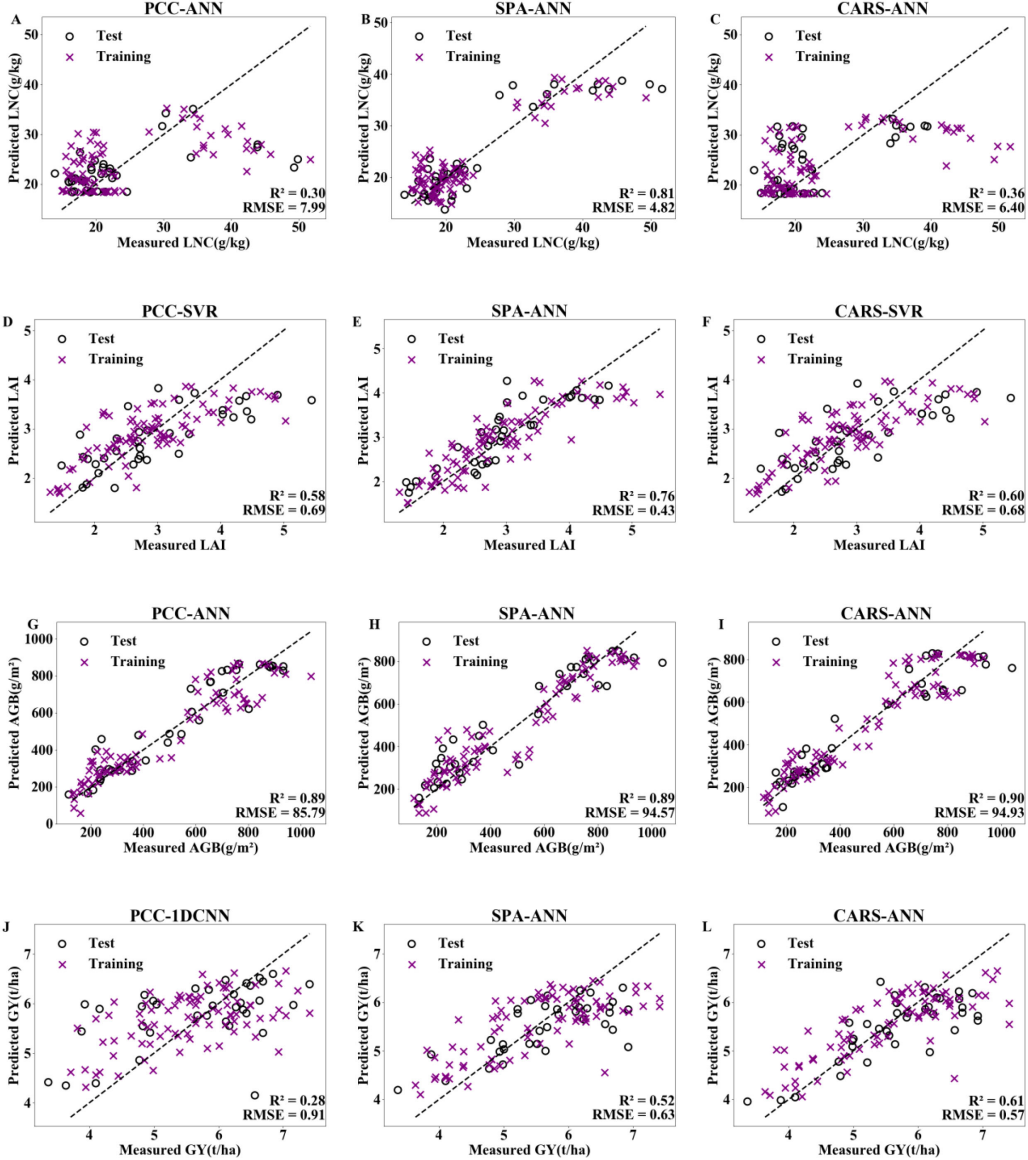
Model validation for LNC **(A–C)**, LAI **(D–F)**, AGB **(H–J)**, and GY **(K–M)** with optimal performance under PCC, SPA, and CARS dimensionality reduction methods combined with machine learning algorithms. LNC, LAI, AGB and GY represent the leaf nitrogen concentration, leaf area index, aboveground biomass and grain yield, respectively. PCC, SPA and CARS represent person correlation coefficient, successive projections algorithm, and competitive adaptive reweighted sampling, respectively. ANN, SVR and 1DCNN represent artificial neural network, support vector regression and 1D convolutional neural network.

The optimal dimensionality reduction method and machine learning algorithm were selected for the four indicators, with 70% of the data used as the training set and 30% used as the validation set for verification. The model validation results are shown in **Figure 7**. The SPA–ANN model had the best performance in estimating LNC and LAI, with R² values of 0.81 and 0.76 and RMSE values of 4.82 g/kg and 0.43, respectively. The CARS–ANN model had the best performance in estimating AGB and GY, with R² values of 0.90 and 0.61 and RMSE values of 94.93 g/m^2^ and 0.57 t/ha, respectively. The results indicate that compared with those of the PCC method, the sensitive bands selected by SPA and CARS are more representative. Compared with SVR, 1DCNN, and LSTM, the ANN algorithm has an advantage in estimating rice LNC, LAI, AGB, and GY.

### 3.7 Optimal combination model with texture features

**Figure 8:**
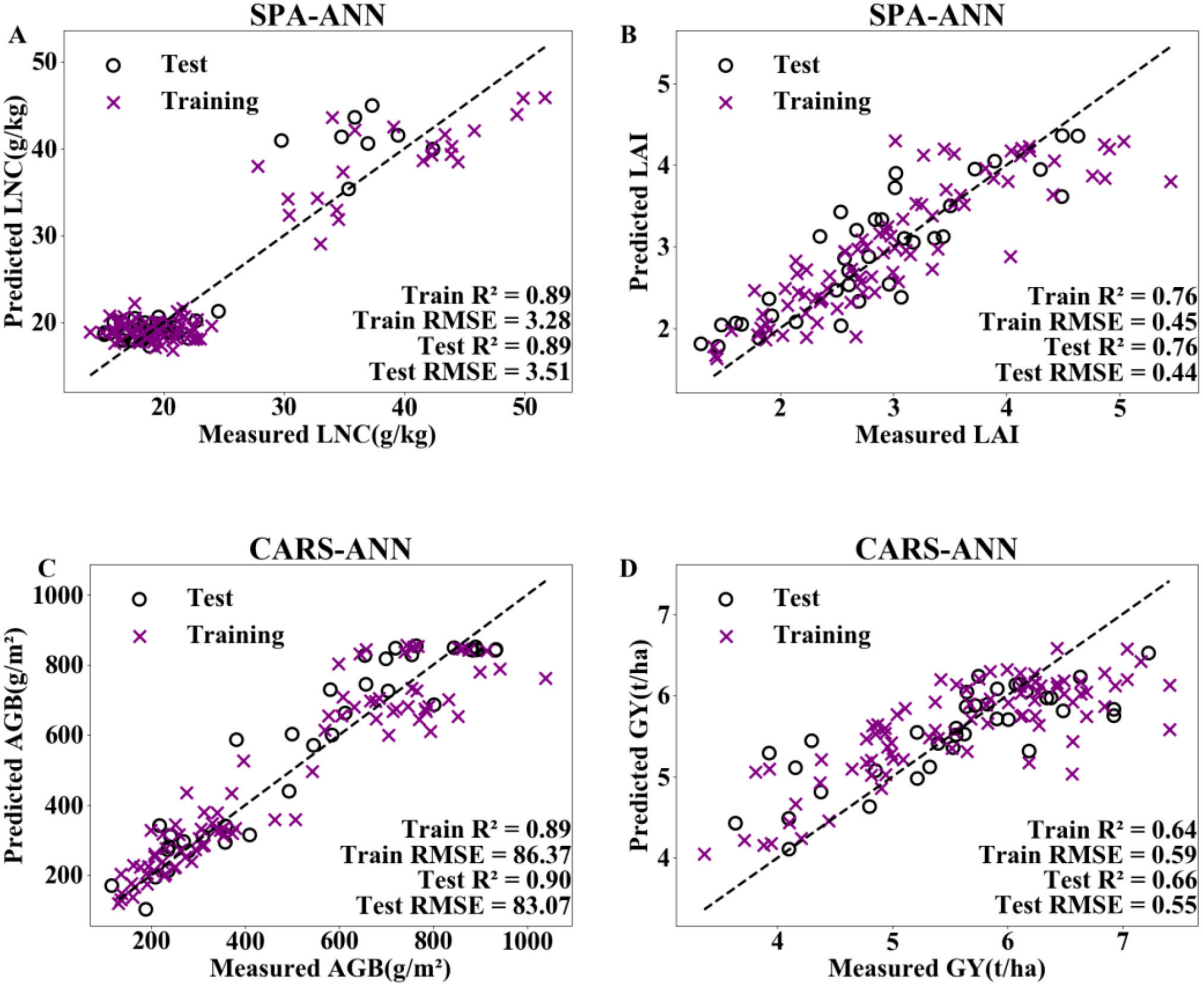
Adding textural features to the optimal combined models for estimating LNC **(A)**, LAI **(B)**, AGB **(C)**, and GY **(D)**. LNC, LAI, AGB and GY represent the leaf nitrogen concentration, leaf area index, aboveground biomass and grain yield, respectively. SPA and CARS represent successive projections algorithm, and competitive adaptive reweighted sampling, respectively. ANN represent artificial neural network.

On the basis of the optimal dimensionality reduction methods and machine learning algorithms for the four indicators in **Figure 7**, texture features corresponding to the sensitive bands were introduced. The results are shown in **Figure 8** and **Table 3**. The models constructed by incorporating texture features all showed certain improvements. In estimating LNC, compared with spectral modelling alone, the R² of the training set increased by 8.5%, and the RMSE decreased by 10.9% when texture features were introduced into the model; the R² of the test set increased by 9.9%, and the RMSE decreased by 27.2%. In estimating GY, the R² of the test set increased by 8.2% and the RMSE decreased by 3.5% when texture features were incorporated into the modelling.

## 4 Discussion

### 4.1 Impact of dimensionality reduction methods on model accuracy

Performing dimensionality reduction on spectral data to reduce redundancy is a key step in establishing crop growth parameter estimation models and is an important aspect of improving model accuracy and stability. The three dimensionality reduction methods, PCC, SPA, and CARS, can all be used to effectively reduce the dimensionality of spectral data and select sensitive bands for specific growth parameters (**Table 2**). These methods have been widely used in numerous studies to extract key spectral features (Zhang et al., 2022; Qin et al., 2020). Che et al. (Che et al., 2024) used PCC to select the three spectral bands with the highest correlation with rice nitrogen: 756 nm, 813 nm, and 899 nm. They conducted nitrogen estimation modelling with these bands, achieving high precision and stability, with an R² of 0.886 and an RMSE of 1.104. Liu et al. (Liu et al., 2024) used SPA to select sensitive bands for classifying rice blast disease. The classification accuracy was second only to that of the PCA method, with a modelling R² of 0.933 and a kappa coefficient of 91.67%. Xu et al. (Xu et al., 2022) used CARS to reduce the dimensionality of spectral data, and the nitrogen estimation model constructed with the selected sensitive bands had R² values of 0.690 and 0.596 and RMSE values of 0.669 and 0.774 mg/g for the training and validation sets, respectively. Therefore, there are also obvious differences in the sensitive bands obtained and in the model prediction performance when different dimensionality reduction methods are used. This is because these dimensionality reduction methods have different emphases on feature selection. For example, the CARS algorithm is similar to the “survival of the fittest” principle in Darwin’s theory (Li et al., 2009), identifying sensitive bands that have a significant impact on the target variable through Monte Carlo model sampling and exponential decay wavelength selection. PCC focuses on the strength of linear correlation and on selecting multiple bands with high correlations, but these bands may contain redundant information, affecting the model’s predictive performance. The SPA selects bands that contribute the most to the model step by step, and it can establish a band combination with the least redundancy while effectively reducing the collinearity among variables (Araújo et al., 2001). In this experiment, when LNC and LAI were estimated, dimensionality reduction by the SPA was significantly better than that via the PCC and CARS algorithms (**Table 3**, **Figure 7**). Changes in LNC can affect the chlorophyll content of plant leaves, which in turn affects leaf spectral reflectance (Gitelson et al., 2003). LAI refers to the leaf area per unit land area. An increase in the LAI means that there is more leaf area, which is likely to increase the scattering and reflection of spectra. Research has shown that an increase in the LAI can lead to a significant increase in spectral reflectance in the near–infrared band (760–1315 nm) (Qin et al., 2017). Li et al. (Li et al., 2003) reported that the extent of the impact of chlorophyll content changes on canopy reflectance is related to the LAI. The larger the LNC and LAI are, the greater the chlorophyll content, and the stronger the canopy reflectance. Therefore, both the LAI and LNC have a direct connection with leaf spectral characteristics. SPA is a forwards iterative search method used to select wavelengths with the least redundant spectral information to solve collinearity problems (Galvo et al., 2008). The algorithm is relatively simple and has high computational efficiency, making it suitable for screening sensitive bands directly related to leaf spectral characteristics. When AGB and GY were estimated, the sensitive bands selected by CARS dimensionality reduction were clearly superior to those selected by SPA and PCC (**Table 3**, **Figure 7**). This is because AGB and GY are related not only to spectral data but also to texture features and spatial structures, among other factors. There are complex interactions among these factors, and this complexity requires that the estimation models be able to handle and interpret these complex multivariate relationships. The CARS algorithm is well– suited for handling complex multivariate relationships and hyperspectral data with a large amount of redundant information and multicollinearity. It can effectively select the key feature bands that contribute the most from among complex data and can reduce the number of data dimensions, thereby achieving higher model accuracy and stability (Tang et al., 2021).

### 4.2 Impact of machine learning algorithms on model accuracy

The results of this experiment indicate that the ANN model performs better than the SVR, 1DCNN, and LSTM models do. The optimal model for all four biological indicators is the ANN model (see **Table 3**, **Figure 7**). ANNs are primarily applied to prediction and classification problems and are among the most widely used machine learning algorithms at present. Among the many machine learning algorithms, it has relatively good accuracy and stability (Wang et al., 2023). SVR is often used to address regression and classification problems in pattern recognition and data analysis (Li et al., 2024). It can avoid the effects of noise generated by data, thereby reducing the risk of model overfitting. However, owing to the need to calculate the kernel function and adjust the parameters, the choice of parameters may have a significant effect on the model’s performance (Liu et al., 2017). 1DCNN is a special type of feedforward neural network used for processing sequential data and is currently one of the more popular deep learning methods. It is mainly used for processing image and speech data (Ye and Zheng, 2024), but it has an inconveniently large number of parameters to adjust, and the interpretability of the results is not very high. LSTM is a special RNN structure that is often used for sequence data analysis. It can better address the problems of gradient vanishing and gradient explosion during the training of long sequences (Li and Cao, 2018), and it performs better when processing longer sequential data. In previous studies, ANN estimation models for various crop physiological parameter indicators all demonstrated good performance, particularly in terms of prediction accuracy, stability, and generalizability (Santos et al., 2021; Krupavathi et al., 2022). Santos et al. (Santos et al.2021) constructed an ANN estimation model for soybean yield, with an R² of 0.88 and an RMSE of 167.85. Krupavathi et al. (Krupavathi et al., 2022) used the ANN algorithm to estimate sugarcane yield, with the R² of the best model reaching 0.867–0.916 on the training set and 0.829–0.991 on the test set. As a universal function approximator, an ANN is nonlinear because of its activation function (Zhang H. et al., 2021), enabling it to learn any complex nonlinear relationship between inputs and outputs. This capability allows it to perform excellently when dealing with nonlinear problems. In comparison, SVR and 1DCNN are less flexible than ANN when dealing with nonlinear datasets. Although LSTM is suitable for processing sequential data, it has disadvantages in computational complexity and parallelization capabilities, which leads to poor performance in estimating the biological indicators of interest.

### 4.3 Impact of adding texture features on model accuracy

When texture features were added to the optimal combination model for optimization, the estimation model for LNC achieved the greatest improvement in accuracy. The R² of the model’s test set increased by up to 9.9%, and the RMSE was reduced by up to 27.2% (**Table 4**, **Figure 8**). Texture is an intrinsic characteristic of the surface of an object, independent of changes in color and brightness, and can be used to address cases such as –different objects with the same spectrum and –one object with multiple spectra (Wang et al., 2009). Cheng et al. (Cheng et al., 2024) reported that after texture features were added, the precision of comprehensive growth models for winter wheat constructed using three machine learning methods improved. This is mainly because texture features provide additional spatial information, which helps capture the microstructures and patterns of objects. Under the experimental conditions, the model accuracies for the LAI, AGB, and GY all improved after texture features were added, but the improvements were limited (**Table 4**, **Figure 8**). This is because the optimal dimensionality reduction + machine learning combination model was used during the research process, and the model accuracy was already high, leaving limited room for further improvement. Furthermore, for the four indicators in this experiment, the AGB estimation model had the highest accuracy (**Tables 2**, **4**, **Figures 7**, **8**). This is because when the AGB is larger, more light energy is absorbed for photosynthesis, resulting in lower reflectance in these bands, making it easier to select sensitive bands. Consequently, greater model accuracy can be achieved when the AGB estimation model is constructed. Casanova et al. (Casanova et al., 1998) obtained similar results. They combined rice spectral reflectance with information on the photosynthesis process to establish rice AGB and LAI estimation models, and their results also showed greater model accuracy and stability for AGB estimation than LAI estimation. Zhang et al. (Zhang et al., 2025) employed the Spectral-Texture Fusion Indices (STFIs) method to estimate rice leaf nitrogen content. This approach deeply integrates selected sensitive spectral bands and key texture features through mathematical operations. Among all feature combinations, the optimal model—SFS-DNN combined with STFIs—achieved an R^2^ of 0.874 and an RMSE of 2.621 mg/g. In future research, the STFI methodology would be referenced to construct novel fusion indices by mathematically combining spectral data with corresponding key texture features, thereby building more robust and accurate estimation models and providing stronger technical support for precision agriculture management.

**Table 4:** Spectral models and spectral + texture models.

Dimensionality reduction methods	Machine Learning Algorithms	LNC		LAI		AGB		GY	
		R²	RMSE	R²	RMSE	R²	RMSE	R²	RMSE
PCC	ANN	0.31	7.63	0.58	0.56	0.89	86.25	0.26	0.79
	SVR	0.28	8.30	0.59	0.56	0.85	99.99	0.28	0.78
	1DCNN	0.27	8.02	0.53	0.62	0.85	98.95	0.29	0.75
	LSTM	0.26	7.81	0.50	0.66	0.73	141.31	0.28	0.91
SPA	ANN	0.82	3.68	0.75	0.47	0.90	81.29	0.54	0.65
	SVR	0.73	5.42	0.67	0.56	0.85	97.02	0.47	0.69
	1DCNN	0.79	4.60	0.68	0.53	0.87	94.57	0.50	0.69
	LSTM	0.28	8.22	0.48	0.66	0.74	130.59	0.40	0.79
CARS	ANN	0.35	8.01	0.61	0.58	0.90	79.05	0.63	0.59
	SVR	0.28	8.27	0.62	0.54	0.87	95.15	0.53	0.67
	1DCNN	0.27	8.01	0.58	0.58	0.84	107.07	0.43	0.72
	LSTM	0.28	7.45	0.48	0.69	0.70	148.19	0.10	0.95

LNC, LAI, AGB and GY represent the leaf nitrogen concentration, leaf area index, aboveground biomass and grain yield, respectively.

## 5 Conclusion

Under the conditions of this experiment, the feature bands obtained after SPA dimensionality reduction were best for estimating LNC and LAI, whereas the feature bands after CARS dimensionality reduction were best for estimating AGB and GY. The estimation models for LNC, LAI, AGB, and GY constructed using the ANN method had higher accuracy and stability than those built via the SVR, 1DCNN, and LSTM models. The SPA–ANN model was optimal for estimating LNC and LAI, whereas the CARS–ANN model was optimal for estimating AGB and GY. The model precision improved when texture features were incorporated into the optimal combination model. The model constructed by LNC through “spectral data + texture data + dimensionality reduction + machine learning” yielded the greatest improvement in precision. These results provide a scientific basis for the –nondestructive real–time prediction of rice yield–related traits and precise diagnosis of phenotypes in indica rice in South China.

## Data Availability

The raw data supporting the conclusions of this article will be made available by the authors, without undue reservation.
